# Alternative reproductive tactics in male freshwater fish influence the accuracy of species recognition

**DOI:** 10.1002/ece3.7267

**Published:** 2021-03-29

**Authors:** Shingo Fujimoto, Kaori Tsurui‐Sato, Naotaka Katsube, Haruki Tatsuta, Kazuki Tsuji

**Affiliations:** ^1^ Center for Strategic Research Project University of the Ryukyus Nishihara Japan; ^2^ Faculty of Agriculture University of the Ryukyus Nishihara Japan; ^3^ The United Graduate School of Agricultural Sciences Kagoshima University Kagoshima Japan; ^4^ Present address: Graduate School of Medicine University of the Ryukyus Okinawa Japan

**Keywords:** Fisherian process, male mate choice, mate recognition, Poeciliidae, reproductive interference

## Abstract

Sexual conflict can result in coercive mating. Because males bear low costs of heterospecific mating, coercive males may engage in misdirected mating attempts toward heterospecific females. In contrast, sexual selection through consensual mate choice can cause mate recognition cues among species to diverge, leading to more accurate species recognition. Some species show both coercive mating and mate choice‐associated courtship behaviors as male alternative reproductive tactics. We hypothesized that if the selection pressures on each tactic differ, then the accuracy of species recognition would also change depending on the mating tactic adopted. We tested this hypothesis in the guppy (*Poecilia reticulata*) and mosquitofish (*Gambusia affinis*) by a series of choice experiments. *Poecilia reticulata* and *G*. *affinis* males both showed imperfect species recognition and directed all components of mating behavior toward heterospecific females. They tended to direct courtship displays more frequently toward conspecific than heterospecific females. With male *P*. *reticulata*, however, accurate species recognition disappeared when they attempted coercive copulation: they directed coercions more frequently toward heterospecific females. We also found that heterospecific sexual interaction had little effect on the fecundity of gravid females, which suggests that prepregnancy interactions likely underpin the exclusion of *G*. *affinis* by *P. reticulata* in our region.

## Graphical abstract

*P. reticulata* and *G. affinis* males both showed imperfect species recognition and directed all components of mating behavior toward heterospecific females. With male *P. reticulata*, species recognition was less accurate when they attempted coercive copulation: they tended to direct coercions more frequently toward heterospecific females.

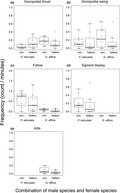

## 1 INTRODUCTION

In polygamous animals, imperfect species recognition by males often results in interspecific mating (e.g., Gröning & Hochkirch, 2008; Hettyey et al., 2014; Russell et al., 2006; Svensson et al., 2007). Even though interspecific mating incurs various fitness costs, such as lower reproductive success, higher mortality, and expenditure of energy and time (Gröning & Hochkirch, 2008; Servedio & Noor, 2003), males often show inaccurate mate recognition and misidentify females of other species as mates. Many researchers have considered that inaccurate mate recognition in males might be related to sexual conflict, in which males and females have differential optima in the mating frequencies (Arnqvist & Rowe, 2005; Bateman, 1948; Parker, 2006). Sexual conflict also can cause competition between males over mating opportunities that can lead to the evolution of coercive male mating behaviors (Parker, 2006). In this circumstance, the males’ cost of mistakenly mating with a wrong individual—that is, a female of another species—is small compared with the possible benefit obtained if the mate were to be a sexually receptive conspecific female (Grether et al., 2017; Takakura et al., 2015). The above scenario suggests that sexual conflict can cause the evolution of imperfect species recognition in male mating behavior.

Another scenario is possible: sexual selection through mate choice can lead to the evolution of accurate species recognition in both females and males. Sexual cues such as male sexual ornaments and female mate preference can coevolve through Fisher's runaway process (Mead & Arnold, 2004). Such Fisherian processes can result in the evolution of accurate species recognition (Servedio & Boughman, 2017; Servedio et al., 2011). Furthermore, several studies provide evidence of male mate choice as well as female mate choice in fishes (Espinedo et al., 2010; Martin & Mendelson, 2016), reptiles (Heathcote et al., 2016), and insects (Chung et al., 2014; Jiggins et al., 2001).

The above two scenarios provide contradictory predictions regarding the accuracy of species recognition in males depending on which was the evolutionary driving force, sexual conflict, or mate choice. Generally, both sexual conflict and mate choice are associated with polygamous mating systems (e.g., Henshaw et al., 2016; Kyogoku & Sota, 2017). Therefore, the two opposing selection pressures can simultaneously operate on species recognition, which may lead to the evolution of an intermediate optimum of species recognition. Therefore, evaluation of the relative importance of the two selection pressures in forming species recognition is an important but difficult challenge in empirical studies.

Here, we focus on alternative reproductive tactics (ARTs), which provide an opportunity to separate two scenarios of male species recognition in empirical studies. ARTs refer to alternative ways to obtain fertilizations (Gross, 1996; Tinghitella et al., 2017). Males of many species are known to show both coercive copulation and courtship displays as ARTs (e.g., Holland & Rice, 1998; Puts, 2010; Wang et al., 2015). Such ARTs are sometimes performed even by the same individual (Taborsky et al., 2008). We hypothesize that the evolutionary driving forces differ between the tactics—that is, sexual conflict leads to coercive copulations, whereas courtship display is associated with mate choice. If this interpretation is correct, the accuracy of species recognition can be regulated in a context‐dependent manner because the cost–benefit relationship may differ between different tactics (e.g., Ord et al., 2011). We predicted that males would show less accurate species recognition when they execute coercive copulation as compared to when they execute courtship display.

In this study, we used freshwater fishes to examine whether or not the accuracy of species recognition in males depends on components of mating behavior. We also addressed the question of whether coercive mating attempts by conspecific and by heterospecific males impose fitness costs on females. We used introduced populations of guppies (*Poecilia reticulata*) and mosquitofish (*Gambusia affinis*). The two fishes share several reproductive characteristics including ovoviviparity, promiscuousness, and the absence of male parental care. More importantly, in Poeciliid fishes males are known to perform not only various courtship displays but also to engage in coercive mating behavior (Rosen & Tucker, 1961; Wang et al., 2015). Males perform a display in front of females to persuade them to mate (Farr, 1980; Houde, 1997; Liley, 1966). The copulation accepted by a female will provide high reproductive success for the male. When females are gravid, they do not accept copulation. In this case, males often attempt coercive copulation (Pilastro & Bisazza, 1999), in which a male follows a female from behind and inserts its genital organ into the female's gonopore without any display (Head & Brooks, 2006; Liley, 1966).

The presence of ARTs is established in guppies, in which copulation attempts not only follow a male display but also involve male coercion (Pilastro & Bisazza, 1999). In contrast, in mosquitofish the presence of male courtship display to attract female interest is less evident. Mosquitofish male mating seems mostly coercive, by just inserting its genital organ after rushing from behind (Deaton, 2008; Peden, 1972, for details see Method). Therefore, we predict that the context (or ART)‐dependence in species recognition is more likely observed in guppies than in mosquitofish.

The two fishes have come into secondary contact (after speciation) in Okinawa, Japan, owing to anthropogenic introduction to control mosquitos and wild release of ornamental fish (Kochi, 1997; Ishikawa et al., 2013; Reznick et al., 2017). *Poecilia reticulata* was introduced to Okinawa in the 1960s and now tends to exclude *G*. *affinis*, which was introduced there in the early 20th century (Kochi, 1997; Tsurui‐Sato et al., 2019). Tsurui‐Sato et al. (2019) demonstrated that an underlying mechanism for this exclusion was reproductive interference; that is, a negative effect on female fitness caused by interspecific reproductive behavior (e.g., Gröning & Hochkirch, 2008). Despite the phylogenetic distance (i.e., different genera), heterospecific mating does occur between the two species in both directions (Tsurui‐Sato et al., 2019). These finding indicate that these introduced populations in Okinawa provide a model system to test our hypothesis on the relationship between the species recognition and ARTs.

Moreover, although the fecundity of *G. affinis* females was reduced by the presence of *P. reticulata* males in a previous study (Tsurui‐Sato et al., 2019), the mechanism remains unclear. The authors stressed that the mechanism during fertilization is important, such as gamete loss due to hybrid lethality. However, reproductive interference can occur by other mechanisms as well. For example, sexual harassment from males led to a 25% decrease in foraging by females (Magurran & Seghers, 1994). Because the fecundity of female fish is a product of their feeding success, a mating attempt by a conspecific or heterospecific male may decrease female fitness (e.g., Magurran & Seghers, 1994; Makowicz & Schlupp, 2013). If the reduction in feeding efficiency is the main mechanism to cause the offspring loss during pregnancy rather than the fertilization process, then gravid females alter their offspring number. Therefore, in this study, we observed the frequency of male mating behavior using gravid females and examined whether the offspring number was lower in the presence of a conspecific or heterospecific male than in a solitary gravid female.

## 2 MATERIALS AND METHOD

### 2.1 Fishes and study sites

The guppy *Poecilia reticulata* originates from Venezuela and South America, and the mosquitofish *Gambusia affinis* is originally from North America (Hrbek et al., 2007; Reznick et al., 2017). According to the ancestral area reconstruction based on the molecular phylogeny of the family Poeciliidae, the genus *Poecilia* diversified mainly in South and Central America, whereas the genus *Gambusia* diversified after it dispersed from Central America to North America (Reznick et al., 2017). *Poecilia reticulata* and *G. affinis* allopatrically evolved over a divergence time estimated at 40 million from a common ancestor. Therefore, natural secondary contact within their native distributions is unlikely to occur. Because interspecific interactions can facilitate the evolution of species recognition regardless of sexual selection, the two species that are not sympatric in the natural distribution provide a good opportunity to examine the ART‐dependence in species recognition.

Although their native distributions do not overlap, both species have been intentionally introduced all over the world for the biological control of mosquitoes (Deacon et al., 2011; Pyke, 2008). The two fishes have come into secondary contact in Okinawa Island, Japan (26°7′N, 127°42′E). *Gambusia affinis* was introduced in Okinawa Island around the early 20th century via Hawaii and Taiwan (Kochi, 1997). The distribution was widespread in Okinawa Island up to the 1960s. However, after multiple introductions of artificially bred lineages of *P. reticulata* in the 1960s (Shoji et al., 2007), *G. affinis* populations reportedly started to be replaced by *P. reticulata* in the late 1970s. At present, the local distribution of the two species tended to be exclusive of each other (see also, Tsurui‐Sato et al., 2019).

All fish used were collected on the main island of Okinawa. *Poecilia reticulata* individuals were collected from a freshwater‐filled ditch located at the Okinawa Prefecture Plant Protection Center (26.21°N, 127.72°E) in May 2016. *Gambusia affinis* individuals were collected from a freshwater‐filled ditch located at the Ginowan Seaside Park (26.28°N, 127.73°E) in May 2016. We chose collection sites where either *P. reticulata* or *G. affinis* was distributed at high density. In these collection sites, the relative abundance of *P. reticulata* (the density of *P. reticulata* divided by the total density of two species) was also highly skewed, less than 2% or more than 98%. Sexual interactions with different species were believed to occur rarely.

Immediately after collection, fishes were transported to the laboratory, where each species was kept separately (at 26 ± 1°C and under natural day length) as a group in glass aquaria (30 × 40 × 60 cm). The maximum density of fish in experimental aquaria (78 fish/m^2^) was lower than in naturally observed limits in Okinawa Island for all experiments. The fish were fed daily with dry fish food at approximately 5% of fish body weight (Hikari Tropical Fancy Guppy; Kyorin, Hyogo, Japan). For each species, we prepared three such stock populations. Fish were allowed to acclimate to laboratory conditions for 1 month prior to the following experiments. Both species have a pregnancy of approximately 30 days (Guevara‐Fiore et al., 2010; Pyke, 2008). Most females in the stock were pregnant. All individuals used for the experiments were sexed based on the shape of their anal fin (Pyke, 2008). Prior to the experiment, gravid females with a wide abdomen were chosen. In *P. reticulata*, females will accept a male's courtship display only when the female is a virgin or immediately after giving birth, and they do not accept males during pregnancy (Houde, 1997). In both *P. reticulata* and *G. affinis*, males can identify female gravid status by olfactory cues (Guevara‐Fiore et al., 2010; Park & Propper, 2002). To minimize the effect of individual differences in female sexual receptivity on male behavior (e.g., Dosen & Montgomerie, 2004; Jeswiet & Godin, 2011), we observed male behaviors toward gravid females.

After the experiments described below, fish were anesthetized with ice and fixed with 5% neutralized formalin for the morphometric studies. We measured the standard length (from the tip of the upper jaw to the caudal fin base) of each individual and measured the wet weight using an electronic balance. Each fish was used only once in the following experiments.

### 2.2 Ethics statement

*Gambusia affinis* is a species that is prohibited under the Invasive Alien Species Act in Japan to be reared or transported in a living condition. We obtained permission for rearing and transportation of *G*. *affinis* from the Ministry of the Environment (Naha Nature Conservation Office, Kyushu Regional Environmental Office, Ministry of the Environment, Government of Japan; No. 15000146). Fieldwork in the Ginowan Seaside Park was permitted by the park management office (Hagoromo Park Management; No. 27, 2017/04/14). Experiments were in compliance with conditions approved by our institute's animal experiment committee (A2017108).

### 2.3 Experiment 1: Male association preference

**FIGURE A1:**
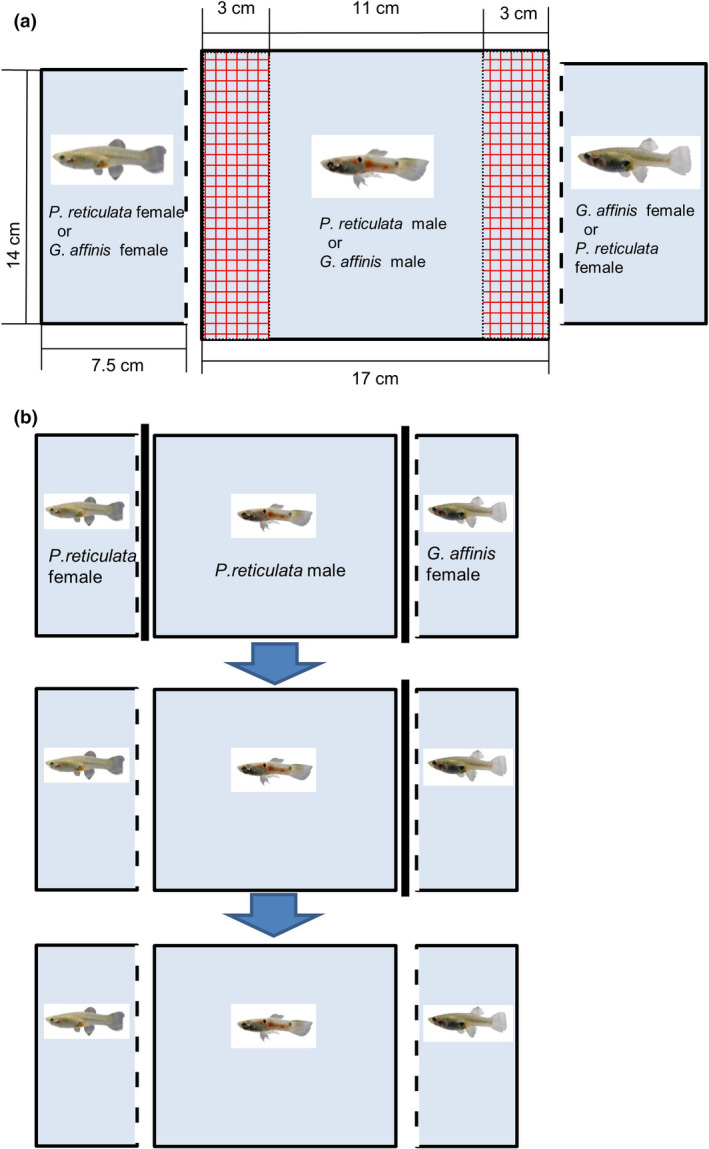
Schema of Experiment 1. (A) Experimental setup; (B) procedure. The marked areas on either side of the center tank in A are the preference zones. The thick vertical lines in B are opaque dividers.

This experiment was designed to test whether or not males of *P*. *reticulata* and *G*. *affinis* exhibit a conspecific mate preference. The experimental setup consisted of a 5 L acrylic aquarium (17 × 17 × 17 cm) placed between two smaller acrylic aquaria (14 × 7.5 × 15 cm) (Figure A1). We defined the 3‐cm‐wide area on each side of the central aquarium as the preference zone (Figure A1). The width of the preference zone was as described by Long and Rosenqvist (1998). We assigned one male in the aquarium and recorded the amount of time the male spent in the conspecific and heterospecific preference zones (defined above), as the association time. Then, we calculated the strength of preference (association time with conspecific or heterospecific female/total association time) for conspecific and heterospecific females.

The procedure of each trial was as follows. In the evening prior to the day of each trial, we randomly chose a male from a stock tank of *P*. *reticulata* or *G*. *affinis*. In both species, because males prefer to mate with larger females (e.g., Deaton, 2008; Dosen & Montgomerie, 2004), size‐matched females (a *P*. *reticulata* and a *G*. *affinis*) were chosen to minimize possible effects of body sizes (within ±0.3 g of wet body weight). The male was placed in the center aquarium that was sandwiched between the two outer aquaria, one with a conspecific female and the other with a heterospecific female. The preference zone closest to the conspecific (heterospecific) female was designated as the conspecific (heterospecific) zone. Until starting the experiment, males and females were visually separated by opaque partitions placed between the aquaria. The next morning (between approx. 09:00 and 11:00 hr), we removed one of the partitions (the conspecific female side) to observe the male sexual activity. During the initial 20‐min acclimation period, some males did not associate with the conspecific female. We regarded those males as sexually inactive and excluded them from the statistical analysis (*P. reticulata*, *n* = 4; *G. affinis*, *n* = 2). After the acclimation period, we removed the other partition (the heterospecific female side) and started the trial. We continuously recorded male behavior for approximately 1 hr using a digital video camera (HDR‐CX180; Sony Corporation, Tokyo, Japan, or HC‐W850M, Panasonic, Osaka, Japan). From those data, the association periods were measured. We replicated this trial for 30 males each of *P*. *reticulata* and *G*. *affinis*.

The male preference for conspecific females was tested using the paired *t* test. The conspecific and heterospecific strengths of preference by a male were treated as a pair. We also examined whether the above preference was affected by the male body weight and the body weight difference between the two females, as discussed in Deaton (2008). The effects of body size on the association time were estimated using a Dirichlet regression model (Maier, 2014), which is based on a framework similar to that of a generalized linear model (GLM) that can be used to analyze a set of proportions (Maier, 2014), such as proportions of time a male spent in the conspecific preference zone, heterospecific preference zone, and intermediate zone between the two preference zones. All statistical analyses in this study were performed using R (version 3.3.2 for Windows, R Core Team, 2016, www.r‐project.org/).

### 2.4 Experiment 2: Male mating behaviors toward conspecific and heterospecific females

This experiment was designed to examine the occurrence of interspecific sexual harassment by males. More importantly, it also aimed to detect differences in male mating behaviors “toward heterospecific females” versus “toward conspecific females.” We assumed that the size of the female or the size difference between the two females affected male mating behaviors (Deaton, 2008; Dosen & Montgomerie, 2004), so we observed male behaviors in a male–female pair to minimize the effect of female size. The female standard length did not significantly differ between species (*t* = −0.33, *p* = 0.74, mean ± *SD* = 25.7 ± 2.5 mm in *P. reticulata* and 26.0 ± 2.1 mm in *G. affinis*). Size‐matched males between species were also chosen to minimize possible effects of body size on mating behaviors (*t* = 1.95, *p* = .06, mean ± *SD* = 20.5 ± 2.4 mm in *P. reticulata* and 19.2 ± 1.2 mm in *G. affinis*). We replicated this experiment eight times for all possible combinations of male species and female species (*P. reticulata* and *G. affinis*), using different individuals, to obtain the balanced design in the statistical analysis.

**FIGURE A2:**
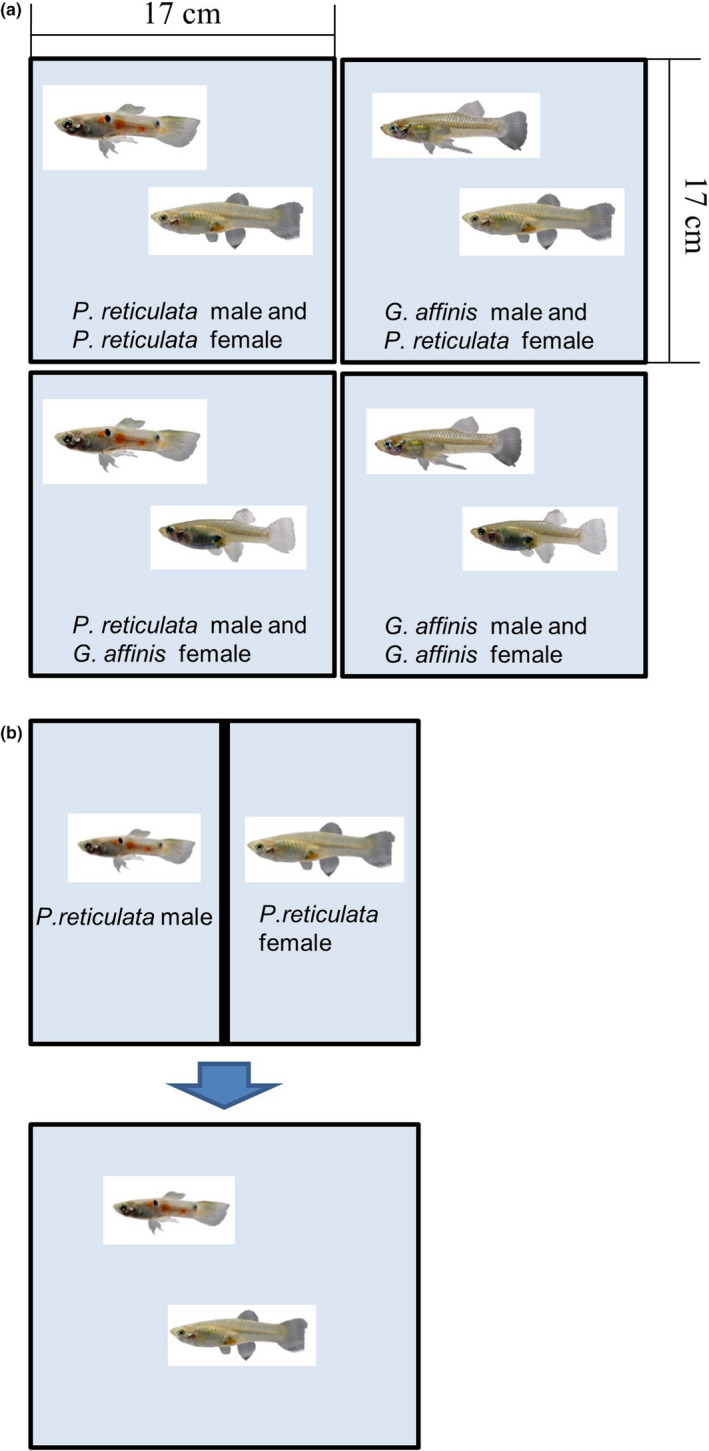
Schema of Experiment 2. (A) Experimental setup; (B) procedure. The thick line in B is an opaque divider.

In the evening on the day before each trial, an individual male *P*. *reticulata* or *G*. *affinis* was placed in a 5 L aquarium (17 × 17 × 17 cm) that also housed a female (one heterospecific female or one conspecific female) (Figure A2). The male and female were separated by an opaque partition inserted in the 5 L aquarium. The next morning (between approx. 09:00 and 10:00 hr) we removed the partition, and the male and female were allowed freely to interact in the aquarium. We recorded their behavior for 20 min using a digital video camera.

We counted the frequency of the five male behaviors defined in the literature on Poeciliid fishes (Krotzer, 1990; Matthews & Wong, 2015; Peden, 1972) toward the female: (1) gonopodial thrust (i.e., the gonopodium is thrust toward the genital region of another individual); (2) gonopodial swing (i.e., the gonopodium is moved away from its resting position underneath the body); (3) follow (i.e., a male follows a female within 1 body length for at least a few seconds); (4) sigmoid display by *P*. *reticulata* male (i.e., a male thrashes forming an “S‐bend” display); and (5) jolts by *G*. *affinis* male (i.e., rapid movement toward the female with shorter duration than “follow”). Note that among the above behaviors, behavior (1) is the most related to coercive copulation in comparison to behaviors (2) to (5). In the preliminary observations, males in both species were sometimes located near females and repeatedly moved their gonopodium without attempting copulation. We treated the gonopodial swing (2) different from the gonopodial thrust (1), with the former regarded as a possible display.

It is established that behavior (4), sigmoid display in the *P. reticulata* males, is related to courtship display (Farr, 1980; Liley, 1966). For the other behaviors examined, however, there seems to be no consensus among researchers if it is categorized coercion or courtship (Liley, 1966; Rosen & Tucker, 1961; Wang et al., 2015). Nevertheless, gonopodial thrust that exists in both species are most reasonably considered more coercive than other behaviors (Deaton, 2008; Farr, 1980; Wang et al., 2015). Thus, we mainly focus on these two distinct male behaviors. For other male behaviors, we quantified its association with the above two behaviors (sigmoid display and gonopodial thrust) by behavioral sequence analysis (the details described Appendix in Figure A4.

To examine the differences in frequencies of each of behaviors (1) to (4) between males and/or females of each species, we used a GLM with negative binomial error (Ver Hoef & Boveng, 2007; Wedderburn, 1974), because these behaviors showed overdispersion in the GLM with Poisson error (Table A1, dispersion parameter, gonopodial thrust 1.62; gonopodial swing 4.82; follow 3.86; sigmoid display 4.83; jolts 1.08). Frequency of behavior (5) jolts was analyzed using a GLM with Poisson error. Males (*P*. *reticulata* or *G*. *affinis*) and the combination of females (conspecific or heterospecific) and their interactions were treated as the fixed effects. This interaction term was absent in the analyses of behaviors (4) sigmoid display and (5) jolts, because those behaviors were each observed in males of only one species. The statistical significance of each factor was tested using the Wald test in a type 1 style analysis (Bolker et al., 2009). The GLM with negative binomial error was performed using the function glm.nb in the package “MASS” (Venables & Ripley, 2002).

### 2.5 Experiment 3: Male effect on female fecundity

**FIGURE A3:**
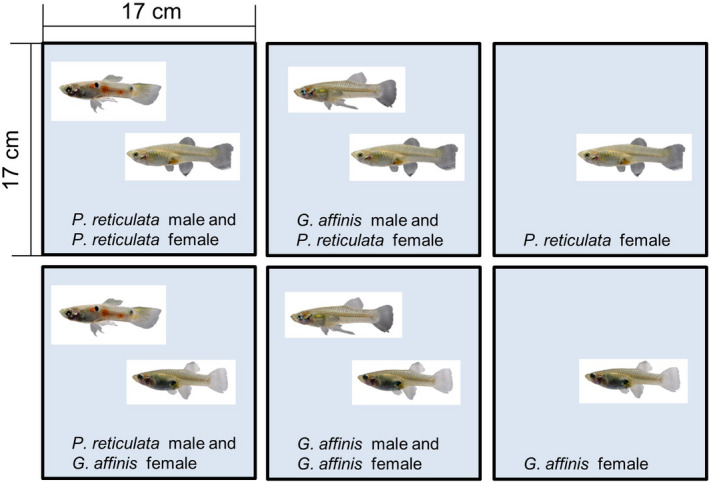
Schema of Experiment 3, experimental setup.

This experiment was designed to examine whether the existence of a conspecific or a heterospecific male influences the number of offspring produced by gravid *P*. *reticulata* and *G*. *affinis* females. In each trial, we put a female with a gravid spot in the 5 L aquarium previously described. This female was either kept alone or with a male of either species (Figure A3). Conspecific males were randomly selected from the same stock tank of the females. The female and the male were allowed to interact freely in the aquarium. Because the duration of this experiment is longer than the previous two experiments, water in the aquarium was continuously circulated and filtered. The fish were fed daily with dry fish food at approximately 5% of fish body weight (Hikari Tropical Fancy Guppy; Kyorin, Hyogo, Japan). The remaining food was removed daily. We checked the fry twice a day to minimize the effect of cannibalism.

We stopped the observation and counted the offspring number when the gravid female released any fry. The date of giving birth was also recorded. The experiment was continued for up to 32 days, considering that pregnancy duration is about 1 month in both species. If the female had released no fry by day 32, we ceased the trial and regarded the fry‐release day to be day 32 and the number of offspring to be zero in the statistical analysis described later. If any fish died, we excluded that trial from the following analysis (for details see Table A2). The number of experimental trials for each treatment group (female alone as the control; with *P. reticulata* male; with *G. affinis* male) was 27, 29, and 9, respectively, in *P. reticulata*, and 15, 9, and 12, respectively, in *G. affinis*. The fry‐release day did not significantly differ between the male treatments (*F*_2,95_ = 0.04, *p* = 0.96) or females in either species (*F*_1,95_ = 1.98, *p* = 0.16). The average (± *SD*) fry‐release day was 13.3 ± 9.4 days in *P. reticulata* and 10.5 ± 9.5 days in *G. affinis*.

We examined whether the presence of a conspecific or a heterospecific male affected female fecundity by using a GLM with Poisson error and log link function. The standard length of the female and the fry‐release day were treated as covariates, and we tested the effect of male species (female alone as a control, with *P. reticulata* male and with *G. affinis* male) using the Wald test. This analysis was conducted separately for the *P. reticulata* female and *G. affinis* female datasets.

## 3 RESULTS

### 3.1 Experiment 1: Male association preference

**FIGURE 1:**
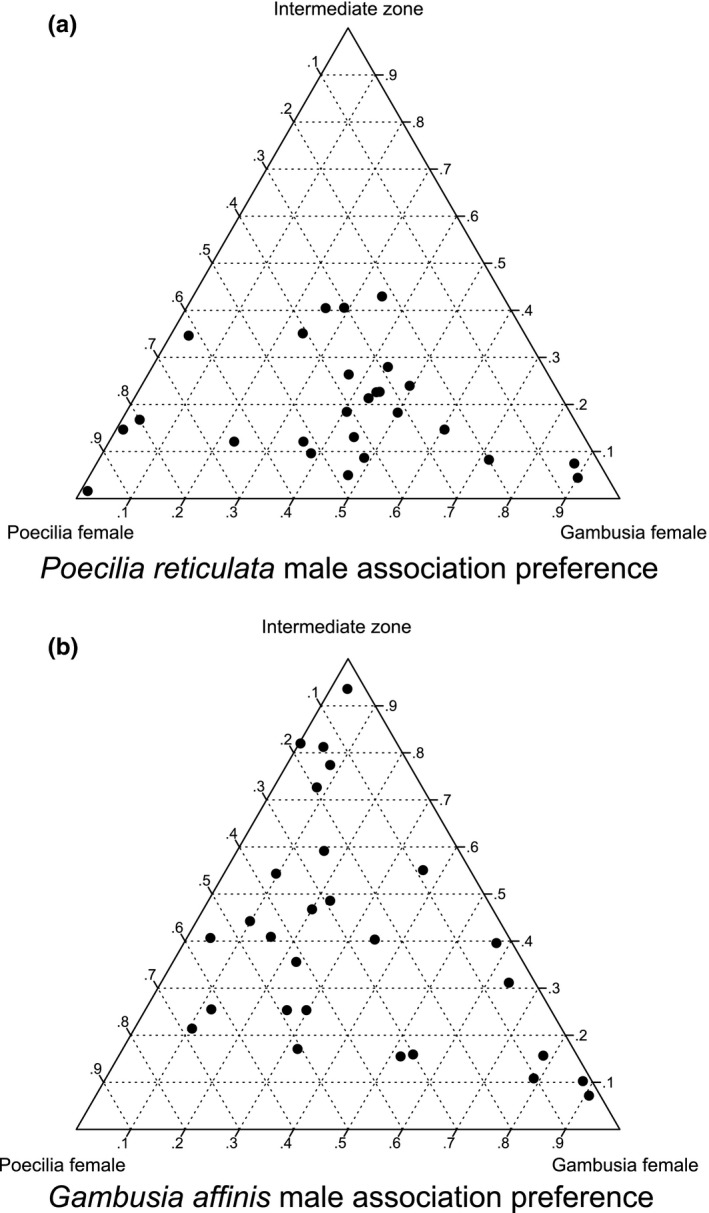
Proportion of association time with *Poecilia reticulata* female and *Gambusia affinis* female (a) Association preference of *P. reticulata* males. (b) association preference of *G. affinis* males

No statistically significant association with conspecific or heterospecific females was detected regardless of the male species (paired *t* tests: *P. reticulata*, *n* = 26, *t* = 0.32, *p* = 0.75; *G. affinis*, *n* = 28, *t* = −0.38, *p* = 0.71). The strength of preference for conspecific females (association time with conspecific female / total association time) was 0.52 ± 0.05 (mean ± *SE*) for *P. reticulata* males and 0.48 ± 0.06 for *G. affinis* males. When focusing on the absolute association time, difference in female body weight did not significantly affect association time, regardless of male species (Table 1). Male body weight had a statistically significant effect on the time spent associating with *G. affinis* females: Smaller males spent less time associating with *G. affinis* females than larger ones regardless of the species of the male. Additionally, males of *G. affinis* tended to spend less time in the preference zones than did males of *P. reticulata* (Figure 1, Table 1).

**TABLE 1:** Results for Dirichlet regression of the proportion of time the male spent in the *Poecilia reticulata* female zone, *Gambusia affinis* female zone, and intermediate zone

Variables	Estimate	*SE*	*Z* value	*p* value
Beta coefficients for: *Gambusia* female side				
Male species	0.46	0.31	1.48	0.14
Body weight of male	13.68	5.46	2.51	0.01*
Female body weight difference	−2.06	1.48	−1.40	0.16
Beta coefficients for: intermediate zone				
Male species	−0.14	0.33	−0.42	0.67
Body weight of male	6.21	4.67	1.33	0.18
Female body weight difference	−2.40	1.56	−1.54	0.12
Beta coefficients for: *Poecilia* female side				
Male species	0.72	0.30	2.40	0.02*
Body weight of male	9.44	5.80	1.63	0.10
Female body weight difference	−1.80	1.45	−1.24	0.22

Note

Values indicate partial regression coefficients and standard errors for each variable, male species (*G. affinis*, *P. reticulata*), body weight of male, body weight difference between females (*P. reticulata* female − *G. affinis* female).

Asterisk showed statistical significance in Wald test (*: *p* < 0.05; **: *p* < 0.01).

### 3.2 Experiment 2: Male mating behaviors toward conspecific and heterospecific females

**FIGURE 2:**
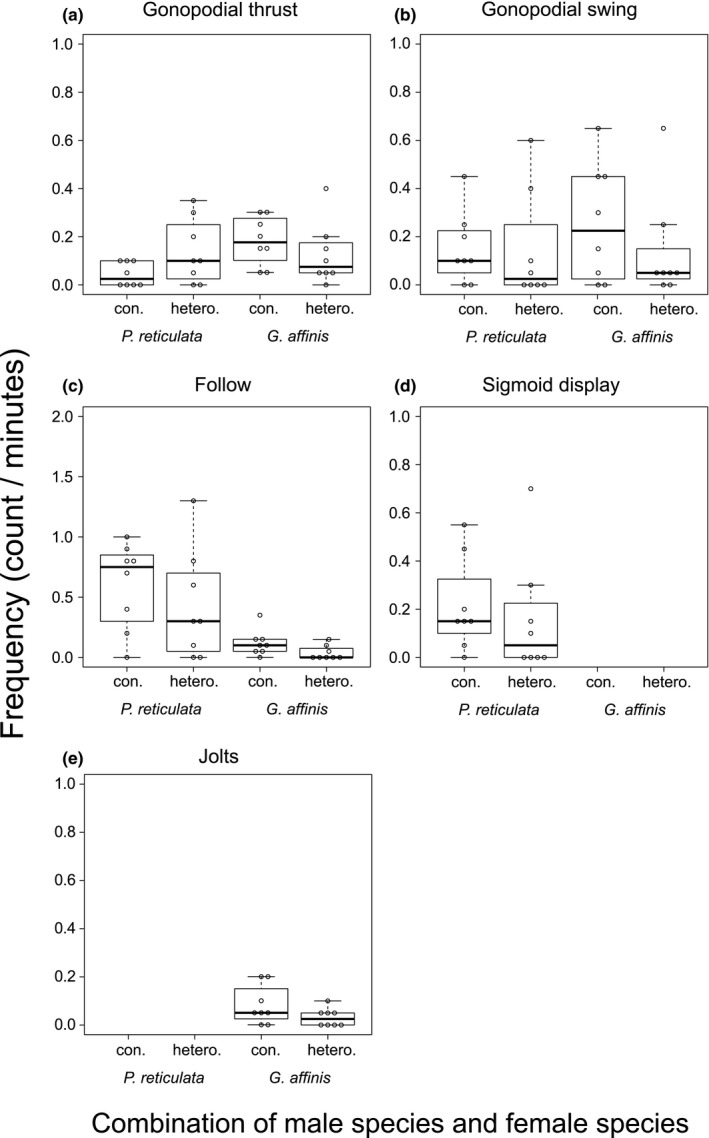
Frequencies of male behaviors toward *Poecilia reticulata* females and *Gambusia* *affinis* females. (a) gonopodial thrust, (b) gonopodial swing, (c) follow, (d) sigmoid display (d), and (e) jolts

**FIGURE A4:**
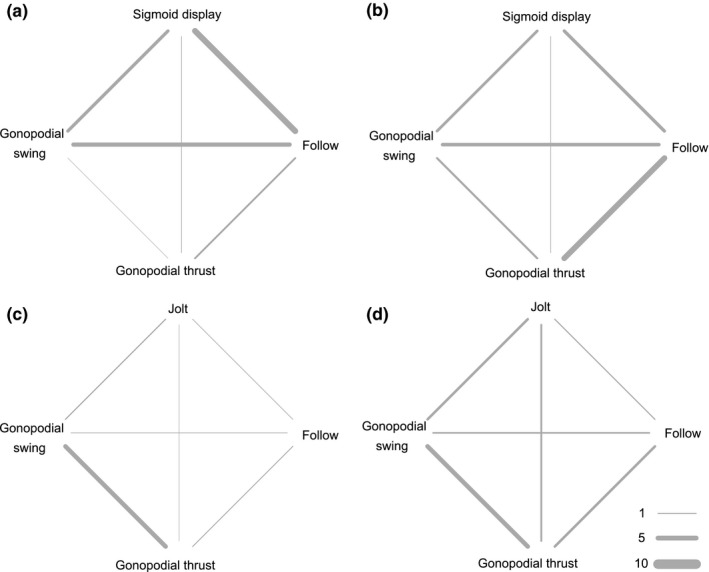
Mating behavior transition network based on the 8 replicates. The line width was corresponded to the average numbers of transition: (A) *P. reticulata* male and *P. reticulata* female; (B) *P. reticulata* male and *G. affnis* female; (C) *G. affinis* male and *P. reticulata* female; (D) *G. affinis* male and *G. affnis* female.

Males of both species performed all of the behaviors (except for sigmoid display and jolts, each of which is specific to males of one species) toward both conspecific and heterospecific females (Figure 2). A statistically significant interaction was detected between male species × female species in the frequency of gonopodial thrust, a behavior related to coercive copulation, in *P*. *reticulata* (Figure 2a, Table 2). Male *P*. *reticulata* tended to perform relatively more coercive toward heterospecific females than did *G*. *affinis* males. Male *P. reticulata* showed a stronger association between follow and gonopodial thrust compared with other combinations of behaviors (Figure A4). This association tended to be stronger in the heterospecific females than in the conspecific females. Compared with the male *P. reticulata*, *G. affinis* males little changed the association of behaviors, regardless of female species.

**TABLE 2:** Results of GLMs with negative binomial errors for the effects of male species, female species, and their interaction on the frequencies of male behaviors

Behavior	Variables	Estimate	*SE*	*Z* value	*p* value
Gonopodial thrust	Male species	−1.42	0.50	−2.84	< 0.01**
	Combination of female species	−0.37	0.40	−0.93	0.35
	Male species × Combination	1.52	0.65	2.34	0.02*
Gonopodial swing	Male species	−0.54	0.67	−0.80	0.42
	Combination of female species	−0.62	0.67	−0.93	0.35
	Male species × Combination	0.58	0. 95	0.61	0.54
Follow	Male species	1.62	0.51	3.14	< 0.01**
	Combination of female species	−1.15	0.65	−1.78	0.08
	Male species × Combination	0.81	0.80	1.00	0.31
Sigmoid display in *P. reticulata* males	Combination of female species	−0.31	0.63	−0.49	0.62
Jolts in *G. affinis* males	Combination of female species	−0.96	0.53	−1.82	0.07

Note

Sigmoid display was analyzed only in *Poecilia reticulata,* and jolts were analyzed only in *Gambusia affinis*, because those were species‐specific behaviors. The other behaviors were analyzed combining data of both species. In the exception, jolts were analyzed a GLM with Poisson error due to no overdispersion (see also Materials and Methods).

Asterisk showed statistical significance in Wald test (*: *p* < 0.05; **: *p* < 0.01).

### 3.3 Experiment 3: Male effect on female fecundity

**FIGURE 3:**
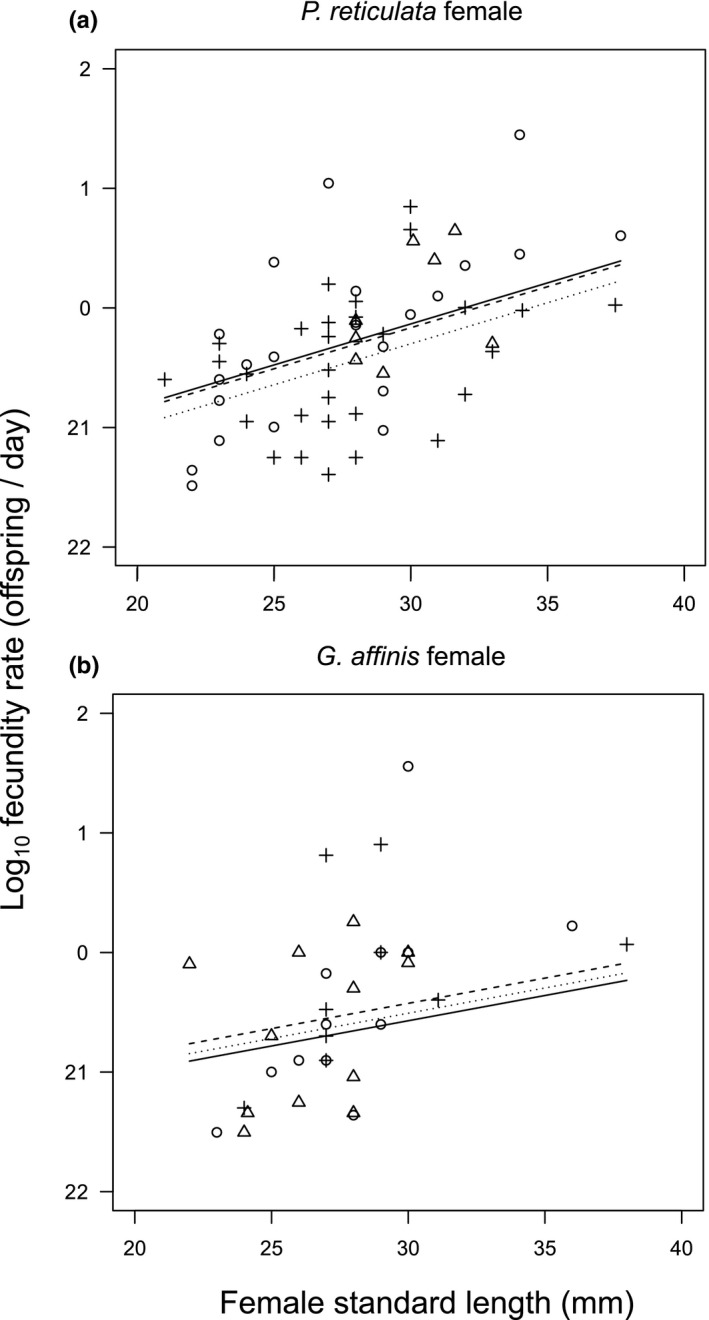
Size–fecundity relationship in (a) *Poecilia reticulata* females and (b) *Gambusia affinis* females. The symbols represent the three treatments (○, control, female alone; +, *P. reticulata* male; △, *G. affinis* male), and the lines represent the prediction from the generalized linear model (solid line, control; dotted line, *P. reticulata* male; dashed line, *G. affinis* male)

Gravid *P*. *reticulata* females released fewer fry when they were housed with a conspecific male, in comparison to the female‐alone condition and those housed with a *G*. *affinis* male (Figure 3a, Table 3). In *G*. *affinis* females, the number of offspring did not differ between the three treatments (Figure 3b, Table 3). Female size positively affected the number of offspring in both female species (*P*. *reticulata* female, estimate ± *SE* = 0.16 ± 0.01, *Z* = 14.15, *p* < 0.01; *G*. *affinis* female, estimate ± *SE* = 0.10 ± 0.02, *Z* = 4.57, *p* < 0.01). The fry‐release day was negatively correlated with offspring number in *G*. *affinis* females but not in *P*. *reticulata* females (*P*. *reticulata* female, estimate ± *SE* = −0.007 ± 0.005, *Z* = −1.27, *p* = 0.20; *G*. *affinis* female, estimate ± *SE* = −0.05 ± 0.01, *Z* = −4.42, *p* < 0.01). The coefficient of determination (*R* ^2^) in the GLM was 0.46 for *P. reticulata* and 0.12 for *G. affinis*.

**TABLE 3:** Results for GLMs with Poisson error of the number of offspring in *Poecilia reticulata* female and in *Gambusia affinis* female in the presence of a conspecific or a heterospecific male

Female species	Variables	Estimate	*SE*	*Z* value	*p* value
*P. reticulata*	Female standard length	0.16	0.01	14.15	<0.01**
	Release days	−0.007	0.005	−1.27	0.20
	*P. reticulata* male	−0.38	0.10	−3.66	<0.01**
	*G. affinis* male	−0.07	0.13	−0.57	0.57
*G. affinis*	Female standard length	0.10	0.02	4.57	<0.01**
	Release days	−0.05	0.01	−4.42	<0.01**
	*P. reticulata* male	0.14	0.20	0.73	0.47
	*G. affinis* male	0.34	0.21	1.56	0.12

Note

Standard length of female and the fry‐release day (the number of days before fry release) were treated as the covariates.

Asterisk showed statistical significance in Wald test (*: *p* < 0.05; **: *p* < 0.01).

## 4 DISCUSSION

### 4.1 Species recognition in the ARTs

We found that species recognition of *P*. *reticulata* and *G*. *affinis* males was imperfect (Figure 1). Males of both species directed all components of mating behavior toward both conspecific and heterospecific females (Figure 2). Similar results were observed when comparing the frequency of these behaviors. Males tended to direct all components of mating behavior more frequently toward conspecific females than toward heterospecific females (Figure 2b–e), but the trend was not statistically significant. This was also the case with male *P*. *reticulata*, with one interesting exception: the gonopodial thrust was performed more frequently toward heterospecific females than toward conspecific females (Figure 2a). We consider that the frequent gonopodial thrust reflects an execution of coercive tactics. In our observation, male *P. reticulata* showed the stronger association between follow and gonopodial thrust toward heterospecific females than toward conspecific females (Figure A4), implying that male *P. reticulata* perform gonopodial thrust without display toward heterospecific females. These results of *P. reticulata* males were in line with our prediction: the accuracy of mate recognition should depend on which of the ARTs—coercion or courtship—the male adopted.

Theoretically, mate choice evolves in the sex that incurs the larger cost per mating event (Edward & Chapman, 2011; Kokko & Johnstone, 2002). The courtship tactics of male *P. reticulata* appears to involve large energetic and opportunity (time) costs. For example, sigmoid display also attracts predators as well as conspecific females (Godin, 1995). When the mating display carries such a mortality cost, males also evolve mate choice (Kokko & Johnstone, 2002). Furthermore, the operational sex ratio (defined as the ratio of sexually mature males to sexually receptive females) may be another factor associated with the evolution of mate choice (Emlen & Oring, 1977). Field data suggest that the sex ratio of guppies tends to be female biased in Okinawa (Table A3). From the above evidence, it is considered that male guppies performing such a costly courtship display should carefully choose the female mating partner so as to gain a benefit that outweighs the large cost of courtship.

In contrast, selection pressure on accurate mate recognition for coercive copulation may be weak because of the absence of display cost. More importantly, sexual conflict leads to minimize the cost of time and energy in male mating events (Edward & Chapman, 2011; Takakura et al., 2015). Males usually discriminate mates based on various sensory information, including visual, odor, and tactile cues. To reduce mate recognition cost, males might use only a part of the information. Such simple mating cues could easily overlap between the species, resulting in inaccurate species recognition (Mendelson & Shaw, 2012).

All *P. reticulata* mating behaviors showed overdispersion in the GLM with Poisson distribution (Table A1), suggesting large individual variation. Previous studies indicated that individual males adopt courtship or coercion in relation to, ornaments (Evans, 2010), female pregnancy status (Guevara‐Fiore et al., 2010), and mating experience of males (Guevara‐Fiore & Endler, 2018). We might have underestimated the accuracy of male species recognition due to the experimental setup, such as our use of gravid females (Guevara‐Fiore et al., 2010; Park & Propper, 2002) and/or the no‐choice design in experiment 2 (Dougherty & Shuker, 2015). Individual male response to both con‐ and heterospecific females is necessary. Further analysis focusing on the relationship between species recognition and individual variation in the ARTs will be needed.

### 4.2 Mate recognition and sexual conflict in *Poecilia reticulata*

*Poecilia reticulata* males directed coercive copulation even more frequently toward the heterospecific females than toward the conspecific females (Figure 2a). Species‐specific mating signals may be involved in this phenomenon. Gravid females of *P*. *reticulata* are known to reject males behaviorally, and males can adjust their mating efforts based on female receptivity (Guevara‐Fiore & Endler, 2018). We consider that *G*. *affinis* females may have no rejection signal that *P*. *reticulata* males can receive and respond to. Indeed, the number of females that clearly showed escape behavior from a male was 50% (8/16) of *P. reticulata* females and 19% (3/16) of *G. affinis* females.

Copulation attempts toward gravid females, implying inaccurate mate recognition can evolve in response to sexual conflict (e.g., Hettyey et al., 2014; Russell et al., 2006). The presence of sexual conflict in *P. reticulata* was also supported from the viewpoint of female fitness. The fecundity of gravid *P. reticulata* females decreased when housed with a conspecific male (Figure 3), as reported previously (Ojanguren & Magurran, 2007). However, in the experiment, the prior number of mates and the time after pregnancy were not controlled. Experiments that account for these factors will clarify the impact of sexual conflict on fecundity.

### 4.3 Reproductive interference between the two fishes

Although Tsurui‐Sato et al. (2019) detected a significant reduction in the population growth rate and decreased fecundity of virgin *G*. *affinis* females in the presence of *P*. *reticulata* males, proximate mechanisms for the reduction in *G. affinis* fitness are still unknown. The current study provides direct behavioral evidence supporting Tsurui‐Sato et al. (2019); that is, the occurrence of misdirected heterospecific coercive copulation between *P*. *reticulata* males and *G*. *affinis* females. An important finding of the current study was that sexual harassment by male *P*. *reticulata* had little effect on the fecundity of gravid *G*. *affinis* females (Figure 3), whereas Tsurui‐Sato et al. (2019) detected a strong negative effect of *P. reticulata* males on the fecundity of virgin *G. affinis* females. In experiment 3, although the total number of *G. affinis* females (*N* = 36) is smaller than that of *P. reticulata* females (*N* = 65), the number of *G. affinis* was comparable to that of our previous study (Tsurui‐Sato et al., 2019). Therefore, it is unlikely that the number of trials is the reason why our experiment did not detect a reduction of fecundity in *G. affinis* females.

These results suggest that reproductive interference may occur during prepregnancy and/or the process of fertilization, such as success rate of sperm transfer and/or gamete loss due to hybrid lethality. However, a question remains unanswered. Experiment 2 showed that both *P. reticulata* males and *G. affinis* males performed heterospecific copulations at comparable high frequencies (Figure 2a). Nevertheless, Tsurui‐Sato et al. (2019) reported that the reproductive interference between the two species was asymmetric, that is, the reproductive interference existed from guppies to mosquitofish but not from mosquitofish to guppies. Further studies are needed to understand the proximate mechanism of asymmetric reproductive interference between the two species.

## 5 CONCLUSION

Sexual conflict and sexual selection provide contradictory predictions regarding the accuracy of species recognition in males. The former predicts the evolution of inaccurate species recognition, whereas the latter predicts accurate species recognition through mate choice involving display. *P. reticulata* males show both coercive and courtship tactic as alternative reproductive tactics (ARTs). Our results demonstrated the accuracy of mate recognition should depend on which of the ARTs—coercion or courtship—the male adopted. *P. reticulata* males show less accurate species recognition when they execute coercive copulation as compared to when they execute courtship display, supporting the relationship between selection mechanisms and species recognition accuracy. The species recognition accuracy can evolve differently among ARTs in a context‐dependent manner under opposing selection pressures.

## CONFLICT OF INTEREST

The authors declare no conflict of interest.

## AUTHOR CONTRIBUTIONS

**Shingo Fujimoto:** Conceptualization (lead); Data curation (equal); Formal analysis (equal); Investigation (equal); Methodology (lead); Software (lead); Writing‐original draft (lead); Writing‐review & editing (lead). **Kaori Tsurui‐Sato:** Data curation (lead); Investigation (equal); Methodology (equal); Project administration (lead); Writing‐original draft (supporting); Writing‐review & editing (supporting). **Naotaka Katsube:** Data curation (supporting); Investigation (supporting); Methodology (supporting). **Haruki Tatsuta:** Formal analysis (supporting); Methodology (supporting); Software (supporting); Validation (supporting); Writing‐review & editing (supporting). **Kazuki Tsuji:** Conceptualization (equal); Funding acquisition (lead); Project administration (supporting); Resources (lead); Writing‐original draft (lead); Writing‐review & editing (equal).

## Data Availability

Data and R scripts used for statistical analysis are available from the Dryad Digital Repository https://doi.org/10.5061/dryad.5hqbzkh2g.

## APPENDIX A

**TABLE A1:** Results for overdispersion test in the GLMs with Poisson error of the frequencies of male behaviors

Behavior	dispersion	*Z* value	*p* value
Gonopodial thrust	1.62	1.75	.04^*^
Gonopodial swing	4.82	2.99	<.01^**^
Follow	3.86	2.54	.01^**^
Sigmoid display in *Poecilia reticulata* male	4.83	1.86	.03^*^
Jolts in *Gambusia affinis* male	1.08	0.29	.39

Note

In the test, males (*P. reticulata* or *G. affinis*) and the combination of females (conspecific or heterospecific) and their interactions were treated as the fixed effects. This interaction term was absent in the analyses of sigmoid display and jolts. The overdispersion test conducted using the “dispersiontest” function in the “AER” (Kleiber & Zeileis, 2008).

**TABLE A2:** Summary of Experiment 3

Female species	Treatment	Total	C	*N*	Reproductive	Reproductive/*N*	Fry number	Fry release day
*P. reticulata*	Control (female alone)	32	3	29	27	0.93	9.5 ± 8.5	13.7 ± 10.6
	*P. reticulata* male	32	2	30	29	0.97	5.9 ± 5.9	10.1 ± 8.5
	*G. affinis* male	13	2	11	9	0.82	11.8 ± 7.9	13.9 ± 8.9
*G. affinis*	Control (female alone)	18	1	17	15	0.88	4.9 ± 9.0	9.5 ± 10.7
	*P. reticulata* male	15	2	13	9	0.69	5.6 ± 4.9	13.3 ± 9.5
	*G. affinis* male	19	5	14	12	0.86	4.2 ± 5.0	8.4 ± 6.7

Note

Total: total number of trials with a *Poecilia reticulata* female or a *Gambusia affinis* female for each treatment.

C: cases excluded from statistical analysis (=number of experiment fish that died).

*N*: number used for statistical analysis (=Total − C).

Reproductive: number of females releasing fry.

Reproductive/*N*: the ratio of reproductive females.

Fry number: mean and standard deviation of the offspring fry number.

Fry release day: mean and standard deviation of the day female released fry.

**TABLE A3:** Adult sex ratio in a population of *Poecilia reticulata* on the main island of Okinawa

Date	Female	Male	Adult sex ratio	*p* value
2016/04/15	133	73	**0.65**	<.0001^**^
2016/05/12	321	129	**0.71**	<.0001^**^
2016/06/16	517	273	**0.65**	<.0001^**^
2016/07/15	328	276	0.54	.04
2016/08/09	637	411	**0.61**	<.0001^**^
2016/09/06	235	176	0.57	.0042
2016/10/25	96	43	**0.69**	<.0001^**^
2016/11/29	321	222	**0.59**	<.0001^**^
2016/12/26	226	214	0.51	.6
2017/01/26	282	242	0.54	.09
2017/02/16	462	277	**0.63**	<.0001^**^
2017/03/22	333	277	0.55	.03
2017/05/02	351	331	0.51	.47

Note

We repeatedly collected fish from the Makiminato River (26.25°N, 127.74°E) on the main island of Okinawa. Every collection was conducted with the same effort (10 min, two persons) using a scoop net (mesh size, 2 mm; capture area, 770 cm^2^; shaft, 60 cm). Collected fish were sexed based on the shape of the anal fin. The numbers of male and female was counted and used to calculate the adult sex ratio (female/(female + male)). Bold values show when the number of females significantly exceeded the number of males; that is, a statistically significant deviation from 0.5 assuming a binomial distribution. The significance threshold of each *p* value was adjusted by Bonferroni correction (^**^ *p* < .00076 (=0.01/13), ^*^0.00076 < *p* < .0038 (=0.05/13)).

## 1

To categorize each behavior as a coercive or courtship, we focus on the association between the gonopodial thrust and other behaviors. Based on the time series of the male individual's record taken experiment 2, we counted the number of transitions between behaviors following the method in Wang et al. (2015). Wang et al. (2015) proposed the novel analysis using Markov chain theory, which assumed that the transitions are independent: the probabilities of transitions to the next behavioral state are influenced by the current state but not by previous states. We constructed 4×4 matrices including all behavioral transitions in each species. The associations between behaviors were shown using a transition network by the "igraph" package (Csardi and Nepusz, 2006). In the figure, we excluded the components looping the same behavior and summed up the diagonal components in the matrix. Thus, different orders of the same behavioral components represented as one line.

Male *P. reticulata* showed a stronger association between follow and gonopodial thrust compared with other behaviors (Figure A4). This association tended to be stronger in the heterospecific females than in the conspecific females. These results suggest the follow in male *P. reticulata* was related to coercive mating. Similarly, in the *G. affinis*, males showed a stronger association with gonopodial swing and gonopodial thrust compared with other behaviors, which suggest the gonopodial swing in male *G. affinis* was related to coercive mating. In the, *P. reticulata* males, however, gonopodial swing was more associated with sigmoid display rather than gonopodial thrust.
